# Synthesis and Characterization of the Ethylene-Carbonate-Linked L-Valine Derivatives of 4,4-Dimethylcurcumin with Potential Anticancer Activities

**DOI:** 10.3390/molecules26227050

**Published:** 2021-11-22

**Authors:** Der-Yen Lee, Hui-Yi Lin, Manickavasakam Ramasamy, Sheng-Chu Kuo, Pei-Chih Lee, Min-Tsang Hsieh

**Affiliations:** 1 Graduate Institute of Integrated Medicine, China Medical University, No. 91, Hsueh-Shih Road, Taichung 40402, Taiwan; deryen.lee@mail.cmu.edu.tw; 2 Research Center for Chinese Herbal Medicine, China Medical University, Taichung 40402, Taiwan; pingababy@yahoo.com.tw (H.-Y.L.); sckuo@mail.cmu.edu.tw (S.-C.K.); 3 School of Pharmacy, China Medical University, Taichung 40402, Taiwan; u106055122@cmu.edu.tw; 4 Chinese Medicinal Research and Development Center, China Medical University Hospital, Taichung 40447, Taiwan; 5 Graduate Institute of Biomedical Sciences, China Medical University, Taichung 40402, Taiwan; 6 Research Center for Cancer Biology, China Medical University, Taichung 40402, Taiwan

**Keywords:** cellular uptake, 4,4-dimethylcurcumin, LC-MS spectroscopy, lung cancer, water solubility

## Abstract

Natural phenolic products from herbal medicines and dietary plants constitute the main source of lead compounds for the development of the new drug. 4,4-Dimethylcurcumin (**DMCU**) is a synthetic curcumin derivative and exhibits anticancer activities against breast, colon, lung, and liver cancers. However, further development of **DMCU** is limited by unfavorable compound properties such as very low aqueous solubility and moderate stability. To increase its solubility, we installed either or both of the ethylene-carbonate-linked L-valine side chains to **DMCU** phenolic groups and produced targeted **1**-**trifluoroacetic acid** (**1-TFA**) and **2**-**trifluoroacetic acid** (**2-TFA**) derivatives. The terminus L-valine of ethylene-carbonate-linked side chain is known to be a L-type amino acid transporter 1 (LAT1) recognition element and therefore, these two derivatives were expected to readily enter into LAT1-expressing cancer cells. In practice, **1-TFA** or **2-TFA** were synthesized from **DMCU** in four steps with 34–48% overall yield. Based on the corresponding LC-MS analysis, water solubility of **DMCU**, **1-TFA**, and **2-TFA** at room temperature (25 ± 1 °C) were 0.018, 249.7, and 375.8 mg/mL, respectively, indicating >10,000-fold higher solubility of **1-TFA** and **2-TFA** than **DMCU**. Importantly, anti-proliferative assay demonstrated that **2-TFA** is a potent anti-cancer agent against LAT1-expressing lung cancer cells NCI-H460, NCI-H358, and A549 cells due to its high intracellular uptake compared to **DMCU** and **1-TFA**. In this study, we logically designed and synthesized the targeted compounds, established the LC-MS analytical methods for evaluations of drug solubility and intracellular uptake levels, and showed improved solubility and anti-cancer activities of **2-TFA**. Our results provide a strategical direction for the future development of curcuminoid-like phenolic compounds.

## 1. Introduction

Plant-derived phenolic compounds such as stilbenoids, lignans, flavonoids and curcuminoids constitute major sources of natural therapeutic agents and have been shown to have wide applications in chemoprevention and degenerative diseases [1,2,3]. These natural products endowed with metabolically unstable functional groups often exhibit moderate-to-high in vitro potency but unobvious in vivo efficacy [4]. The discrepancies between the in vitro and in vivo effects mainly stem from phenolic functionalities which are highly prone to form phenol glucuronide and sulfate conjugates by the phase II metabolic enzymes [5]. Therefore, structural modifications on the phenol group are potential strategies for further drug development. 4,4-Dimethylcurcumin (**DMCU**) is a synthetic derivative of curcumin via the di-methylation at the 4-position of linear 3,5-dione-containing chain of curcumin [6].

The previous structure–activity relationship (SAR) analysis on curcuminoid derivatives showed that disconnection of the highly conjugated enol system exerted positive effects on the stability and pharmaceutical activities [7]. Hence, **DMCU** exhibited improved anticancer activity against the MCF-7 breast cancer, HCT-116 colon cancer, A549 lung cancer and HepG2 liver cancer cell lines than curcumin [8], conceivably due to the greater reactive oxygen species producing activity and free radical scavenging activity compared to curcumin [9].

The overexpression of esterase, which functions with ester groups, is generally observed in several cancer cells [10]. Recently, we discovered a series of unique bis(hydroxymethyl) alkanoate derivatives of curcuminoids as efficacious anticancer agents [11]. These curcuminoid derivatives could induce G_2_/M phase arrest, autophagy, mitochondrial-associated apoptosis and ER-stress-induced apoptosis in human breast adenocarcinoma MDA-MB-231 cells [12]. Among these synthetic analogs, 4,4-dimethyl substituted curcuminoid 2,2-bis(hydroxymethyl)propionate (**DMCU-HMP**), can be structurally regarded as an ester derivative of **DMCU** and showed potential anti-cancer activities against MDA-MB-231 breast cancer and HCT-116 colon cancer cell with the IC_50_ values of 2.2 and 3.1 μM, respectively. **DMCU-HMP** is expected to convert into **DMCU** in vivo because the 2,2-bis(hydroxymethyl) propionate group of **DMCU-HMP** could readily cleave upon treatment with a considerable amount of esterase that usually exists in plasma, gastrointestinal tract, and liver, respectively. Shortly thereafter, our pharmacokinetic evaluations of **DMCU-HMP** illustrated that **DMCU** appeared in serum of mice after oral administration of **DMCU-HMP** and both **DMCU-HMP** and **DMCU** are contributors to in vivo antitumor efficacy [13]. A formulation study of **DMCU-HMP** is still ongoing in our lab with the intention of increasing oral bioavailability. In light of these promising anticancer effects and predictable high intrinsic clearance of **DMPU**, we attempted to develop alternative drug substances of **DMCU-HMP** by the modification of **DMCU** as anticancer agents against lung cancer. L-type amino acid transporter 1 (LAT1, also known as SLC7A5) belongs to a member of the solute carrier (SLC) family that can mediate the transportation of endogenous and exogenous bio-substances including amino acids, sugar molecules, biologically essential metal cations, fatty acids, neurotransmitters and pharmaceutical drugs across the cell membranes [14,15]. LAT1 functions as a sodium-/pH-independent transporter and preferably import branched-chain amino acids (BCAAs) such as L-leucine, L-isoleucine, L-valine, L-tyrosine and L-tryptophan into cells [16,17].

Recent studies demonstrated that the LAT1 expression in lung cancer cells was significantly higher than in normal cells [18]. The overexpression of LAT1 cause enhanced nutrient absorption that is essential to rapid tumor growth in lung cancers. Inhibition of LAT1 retarded the amino acid uptake and mTOR phosphorylation, suggesting that LAT1 may act as a diagnostic biomarker or therapeutic drug target [19,20]. Recent studies indicated that treatment of anticancer drug molecules linked with the branched-chain amino acid promoiety as LAT1 recognition elements such as L-valine [21], L-tryptophan [22], and L-leucine [23], caused higher LAT1-mediated drug cellular uptake and greater anticancer activity than their parent compounds. The low solubility and poor metabolic stability of **DMCU** are still unfavorable to drug discovery. It was then anticipated that incorporating BCAAs into **DMCU** can have beneficial impacts on the drug solubility and intracellular uptake, leading to potent antiproliferative activity in LAT1-expressing cancer cell.

Here, we developed the targeted derivatives by installing either or both of the ethylene-carbonate-linked L-valine side chains to **DMCU** and characterized their solubility, stability, and antiproliferative activity LAT1-expressing lung tumor cells.

## 2. Results and Discussion

### 2.1. Synthesis

**Figure 1:**
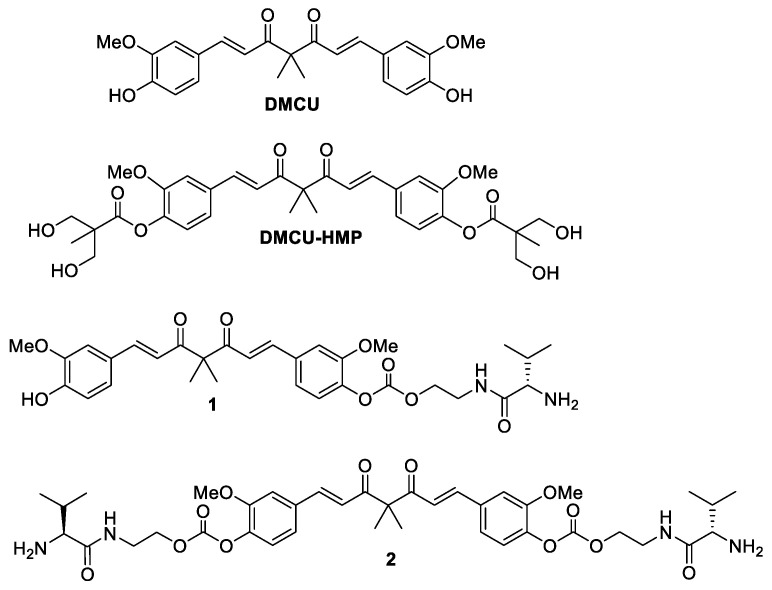
Structure of DMCU, DMCU-HMP, 1 and 2.

**DMCU** with a direct linkage of BCAA to sterically congested phenol group may restrict the close proximity of LAT1 and lower down the enzyme recognition. We anticipated that the introduction of a self-immolating linker between the phenol group and BCAA can tackle the above problem [24]. In the regard, the target derivative **1** was prepared through the addition of a two-carbon side chain to the **DMCU** phenol group via the connection of a carbonate functionality (Figure 1). The *N*-terminus of the two-carbon side chain was linked to a BCAA by an amide bonding. We selected L-valine as the BCAA source since the previous metabolomic analysis indicated that L-valine was increased in the tumors of NSCLS patients, implying NSCLCs prefer to acquire L-valine [25]. To improve LAT recognition, compound **2** was designed as a di-substituted derivative incorporating the ethylene-carbonate-linked L-valine chains on both of the ***DMCU*** phenol groups.

**Scheme 1:**
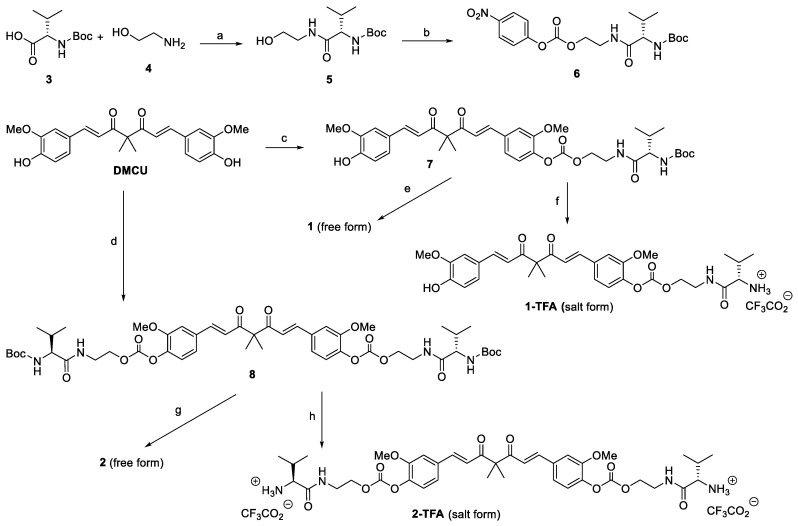
Reagents and conditions: (**a**) HOTT, Et_3_N, CH_3_CN, rt, 3.5 h, 82%; (**b**) 4-nitrophenyl chloroformate, DIPEA, CH_2_Cl_2_, rt, 4 h, 87%; (**c**) **6** (0.9 equiv), Cs_2_CO_3_, DMF, rt, 3.5 h, 48%; (**d**) **6** (2.7 equiv), Cs_2_CO_3_, DMF, rt, 6 h, 68%; (**e**) HCl, CH_2_Cl_2_, rt, 3.5 h then Et_3_N; (**f**) TFA, CH_2_Cl_2_, 4 h, rt, >95% (**g**) HCl, CH_2_Cl_2_, rt, 3.5 h then Et_3_N; (h) TFA, CH_2_Cl_2_, 4 h, rt, >95%.

**Figure 2:**
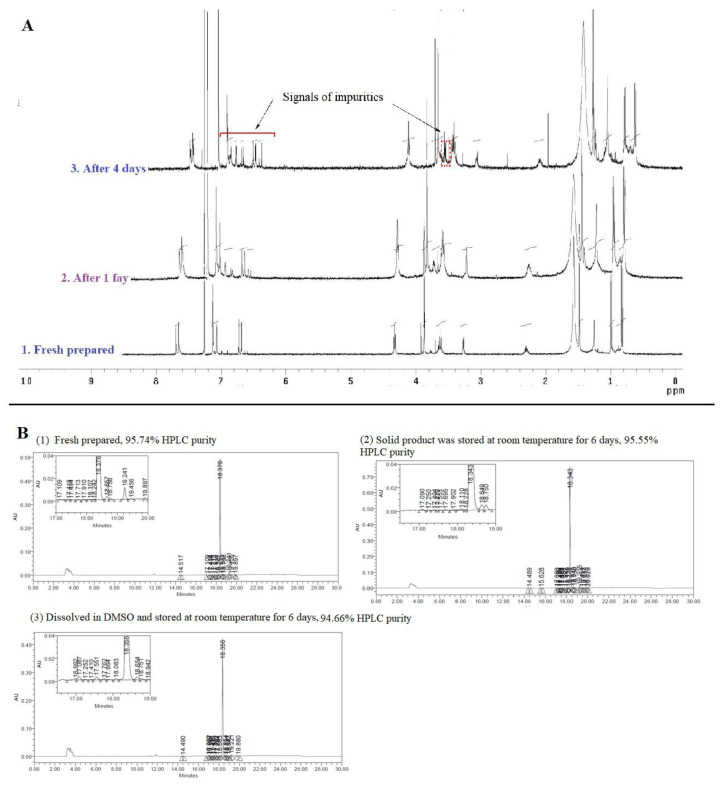
The stability assessments of **2** and **2-TFA**. (**A**) A clear solution of **2** in *d*-chloroform was subjected to a purity assessment by ^1^H NMR spectroscopy at various storage times (fresh prepared (≤1 h); 1 day; 4 days). (**B**) The HPLC purity of fresh prepared **2-TFA** was 95.74%. The fresh prepared **2-TFA** in a solid state was stored at room temperature for another 6 days and then dissolved in water for HPLC analysis. The HPLC purity was decided as 95.55%. The fresh prepared **2-TFA** was dissolved in DMSO and the resulting solution was stored at room temperature for another 6 days. HPLC purity of **2-TFA** in the DMSO solution was 94.66%.

The procedures employed for the synthesis of targeted compounds ***1*** and ***2*** are depicted in Scheme 1. The required **DMCU** was prepared according to a three-step sequence reported in the literature [26]. Initially, the commercially available Boc-L-valine ***3*** was reacted with 2-aminoethanol ***4*** in the presence of HOTT and Et_3_N to provide the amide product ***5*** in 82% yield. Under treatment with 4-nitrophenyl chloroformate and DIPEA, ***5*** was converted into ***6*** in 87% yield. One equivalent or excess amount of **6** was coupled with the deprotonated **DMCU**, formed in situ by the treatment of **DMCU** with Cs_2_CO_3_, to afford compound **7** and **8**, respectively. Compound **7** and **8** thus obtained were subjected to Boc deprotection under the standard conditions to give **1** and **2**, respectively. The preliminary stability test was performed with NMR by applying **2** as a typical example. As shown in Figure 2A, **2** gradually degraded into unidentified products under room temperature as the storage time increased. Based on our previous experience in the synthesis of **DMCU-HMP**, the instability could be ascribed to the cross-hydrolysis of carbonate group by the terminal amino group of side chain. Salt formation reactions of the *N*-terminal L-valine were then carried out to cap the nucleophilic amino group. Between the resulting trifluoroacetic acid (TFA) salt and HCl salt of compound **2**, the stability of TFA salt (**2-TFA**) was greatly improved compared to its free form. The HPLC analysis (Figure 2B) indicated that solid **2-TFA** can be stored in a closed vial under room temperature for 6 days without loss in purity. **2-TFA** is also stable in DMSO solution (0.1 M concentration). After 6-day storage at room temperate, the purity of **2-TFA** remained intact. Accordingly, a similar salt formation procedure was employed to produce the stable **1-TFA**. Compound **1-TFA** along with **2-TFA** were then applied for the following evaluations.

### 2.2. Water Solubility Analysis of ***DMCU***, ***1-TFA***, and ***2-TFA***

**Figure 3:**
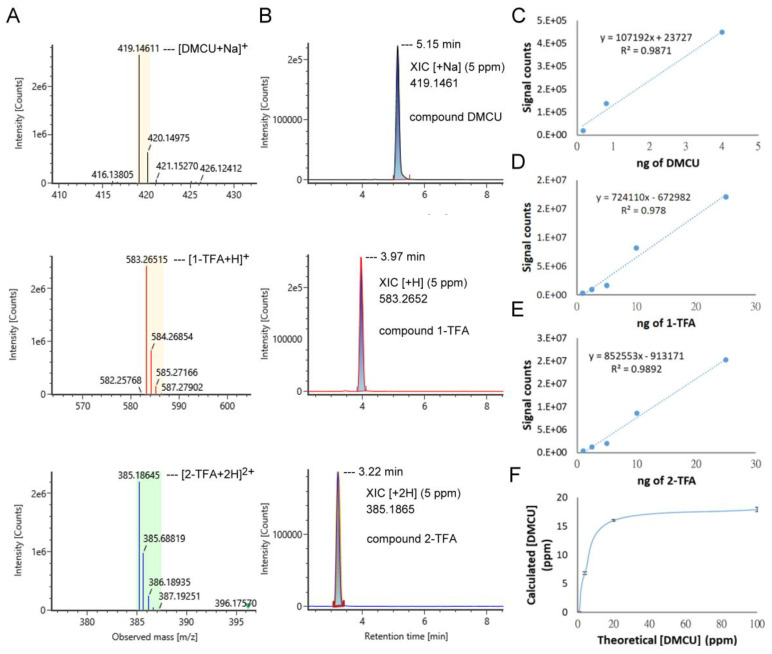
Quantification of water solubility of **DMCU**, **1-TFA**, and **2-TFA**. (**A**,**B**) Mass spectrum (**A**) and extracted ion chromatograms (XIC) (**B**) of **DMCU**, **1-TFA**, and **2-TFA**. (**C**–**E**) Calibration curves of **DMCU (C)**, **1-TFA** (**D**), and **2-TFA** (**E**). (**F**) An investigation on the water solubility of **DMCU**. The solubility of all compounds was determined at room temperature.

As described above, **DMCU** showed an unfavorable drug-like property due to its poor solubility. **DMCU** is virtually insoluble in water and only partially dissolved in some hydrophilic solvents, such as methanol and DMSO. **1-TFA**, and **2-TFA** with the linkage of L-valine-TFA side chain on either or both of the phenol groups were expected to show an increment of water solubility. We used LC-MS analysis to determine the water solubility of **DMCU**, **1-TFA**, and **2-TFA**. Initially, the LC signals detected by ESI+ mode for each compound were assigned as follows: [M + Na]^+^ for **DMCU**, [M + H]^+^ for **1-TFA**, and [M + 2H]^2+^ for **2-TFA** (Figure 3A). The extracted ion chromatogram (XIC) was acquired by extracting the *m*/*z* signal of 419.1461 for **DMCU**, 583.2652 for **1-TFA**, and 385.1865 for **2-TFA** with a tolerance of ±5 ppm for each sample. The retention time of signals obtained from reverse phase liquid chromatography (RPLC) was 5.15 min (**DMCU**), 3.97 min (**1-TFA**), and 3.22 min (**2-TFA**) (Figure 3B). The limit of quantification was 0.16 ng for **DMCU** (S/N = 375.81) and 1 ng for **1-TFA** (S/N = 231.24) or **2-TFA** (S/N = 131.19). Compared to **DMCU**, the shorter retention time of **1-TFA** or **2-TFA** suggested that installation of the ethylene-carbonate-linked L-valine chain resulted in enhancement of hydrophilicity. Next, to determine the water solubility of **DMCU**, **1-TFA** and **2-TFA**, aqueous solutions of these compounds at different concentrations were prepared for the generation of corresponding calibration curves.

The quantitative analysis was performed using LC-MS and the results were shown in Figure 3C–E. Corresponding saturated water solutions were prepared and then diluted with ultrapure water for LC-MS analysis. Using interpolation of signal counted to the corresponding calibration curves, the solubility was decided as 249.7 mg/mL (**1-TFA**) and 375.8 mg/mL (**2-TFA**) at room temperature. For the analysis of **DMCU** water solubility, a clear solution of **DMCU** in 40% MeOH and 60% water (with a concentration of 1000 ppm) was prepared and divided into four portions. Each part was diluted with an appropriate amount of ultrapure water, resulting in **DMCU** precipitates, and the precipitates were then removed by centrifugation to give four samples with theoretical concentrations of 0.8, 4, 20, 100 ppm, respectively. The calculated concentrations of these three samples were decided by comparing the LC-MS signals of corresponding samples to the calibration curves. (Figure 3F). The maximum concentration of **DMCU** in water was confirmed as 18 ppm at room temperature. These data indicated 13,700- and 20,800-fold increased solubility of **1-TFA** and **2-TFA**, respectively, compared to **DMCU** and supported our hypothesis that the chemical modification by the linkage of L-valine-TFA side chain on **DMCU** greatly improved the water solubility.

### 2.3. Anti-Proliferative Evaluation of ***1-TFA***, ***2-TFA***, and ***DMCU*** in LAT1-Expressing Lung Cancer Cells

To evaluate anti-cancer activities of **1-TFA** and **2-TFA**, these two compounds and their reference compound **DMCU** were subjected to the anti-proliferative assay in human non-small-cell lung cancer cell (NSCLC) lines including NCI-H460, NCI-H358 and A549 cell lines (Table 1). As shown, the IC_50_ values of **DMCU** in these cell lines were 1.62–2.50 μM. Compound **2-TFA** with two L-valine containing motifs showed the best anti-proliferative activity among three compounds in all of the NSCLC cell lines we tested. Moreover, compound **1-TFA** with single ethylene-carbonate-linked L-valine chain showed stronger anticancer activity than its parent compound **DMCU** in NCI-H460 cells, but not in NCI-H358 and A549 cells. Interestingly, previous studies showed that L-type amino acid transporters (LAT1) proteins is an amino acid carrier overexpressed in NCI-H460, NCI-H358 and A549 NSCLC cell lines [27,28]. We hypothesized that the instillation of L-valine containing side chains on **DMCU**, like **2-TFA**, may not only greatly improve water solubility but also enhance cellular uptake and promote anti-cancer activity in NSCLC cells with LAT1 overexpression.

### 2.4. Evaluation of Intracellular Uptake and Bioconversion of ***DMCU***, ***1-TFA***, and ***2-TFA*** in NCI-H460 Cell Line

**Figure 4:**
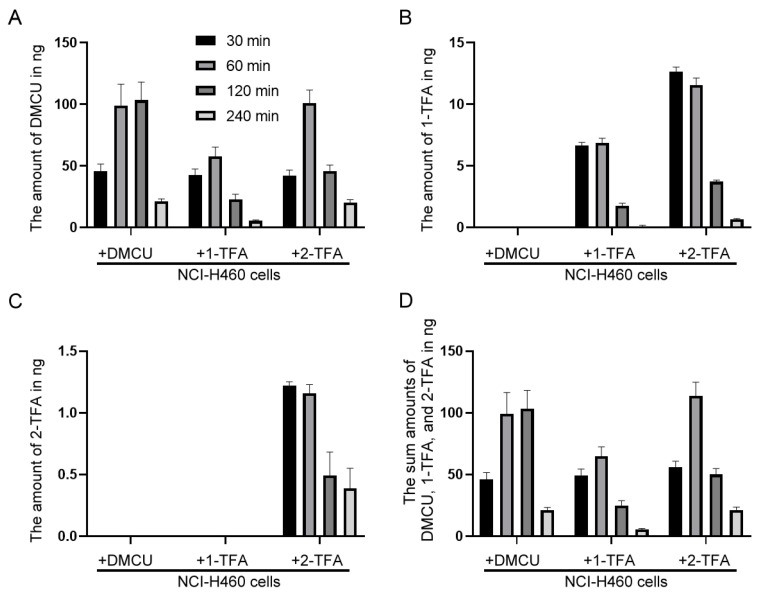
The intracellular levels of **DMCU**, **1-TFA** and **2-TFA** in the NCI-H460 cells. The cellular extracts were obtained from **DMCU**, **1-TFA**, or **2-TFA** treated cells at the indicated time intervals. The amount of drug uptake, (**A**) **DMCU**, (**B**) **1-TFA**, (**C**) **2-TFA**, or (**D**) sum amounts of **DMCU**, **1-TFA**, and **2-TFA**, was evaluated in the cellular extracts of approximately 1 × 106 cells by LC-MS analysis. Data are presented as mean and SD from three experiments.

To test our hypothesis, intracellular uptake of **DMCU**, **1-TFA**, and **2-TFA** were evaluated in NCI-H460 cells which cells were more sensitive to **1-TFA** and **2-TFA** than **DMCU**. The intracellular drug uptake was generally evaluated using a fluorescence microscope or flow cytometry [29,30]. However, the extremely similar chromophores among **DMCU**, **1-TFA**, and **2-TFA** caused failure in detection of individual signals by these two methodologies and therefore drew us to develop a versatile LC-MS based analysis for the cellular uptake evaluations. To estimate the cellular uptake, NCI-H460 cells were treated with each compound for the indicated time periods and the cell extracts were then subjected to LC-MS analysis to determine the intracellular levels of **DMCU**, **1-TFA**, and **TFA** (Figure 4). In the treatment groups, the intracellular levels of each compound were detected by LC-MS where the amount of the compound was quantified by comparing the LC-MS signals of cellular extracts to the corresponding drug calibration curves. Accordingly, the maximal uptake of **DMCU** with a value of about 100 ng was achieved at 1–2 h post-drug treatment (Figure 4A). In **1**-**TFA** treated group, both **1**-**TFA** and **DMCU** were detectable, suggesting that the bioconversion of **1**-**TFA** to **DMCU** proceeded readily in the cells. The maximal uptake levels of **1**-**TFA** and **DMCU** were detected by 60 ng and 7 ng, respectively, at 60 min after treatment (Figure 4A,B). In **2**-**TFA** treated group, the maximal cellular uptakes occurred during the time interval from 30 to 60 min after drug treatment and acquired 100 ng of **DMCU**, 12 ng of **1-TFA** and 1.2 ng of **2-TFA** (Figure 4A–C). All these data implied that the bioconversion of **2-TFA** happened to produce **1-TFA**, which further converted to **DMCU** in the cells. The cellular uptake efficiency of H460 cells was then evaluated in terms of the total quantity of **DMCU**, **1-TFA**, and **2-TFA**. Total drug accumulation in **2-TFA**-treated group was higher than that in **DMCU**-treated and **1-TFA**-treated groups at 30 and 60 min after treatment (Figure 4D). These results indicated that **2-TFA** were absorbed, accumulated in the NCI-H460 cancer cells and the intracellular **2-TFA** were subsequently converted to **DMCU** and **1-TFA** and therefore, treatment of **2-TFA** exhibited stronger anti-cancer effects than **DMCU** and **1-TFA**.

## 3. Materials and Methods

### 3.1. General Synthetic Methods

The reactions were performed under an air atmosphere unless otherwise stated. All solvents and reagents were employed as received. Analytical thin-layer chromatography (TLC) was performed on SiO_2_ 60 F_254_ plates and flash column chromatography was carried out using SiO_2_ 60 (particle size 0.040–0.055 mm, 230–400 mesh), both of which are available from E. Merck (Darmstadt, Germany). Visualization was performed under UV irradiation at 254 nm followed by staining with aqueous potassium permanganate [KMnO_4_ (3 g) and K_2_CO_3_ (20 g) in 300 mL of H_2_O containing 5 mL of an aqueous solution of NaOH (5%, *w*/*v*)] and charring by heat gun. ^1^H and ^13^C NMR spectra were recorded on 500 or 400 FT NMR (Bruker, Billerica, MA, USA). Copies of ^1^H, ^13^C NMR and mass spectra of the synthetic products (**5**, **6**, **7**, **8**, **1-TFA** and **2-TFA**) could be found in Supplementary Materials. Chloroform-*d*, methanol-*d* and *d*-DMSO were used as solvents and TMS (*δ* = 0.00 ppm) as an internal standard. Chemical shifts are reported as *δ* values in ppm as referenced to TMS. Multiplicities are recorded as s (singlet), d (doublet), t (triplet), q (quartet), quint (quintet), sext (sextet), sept (septet), dd (doublet of doublets), dt (doublet of triplets), br (broad), m (multiplet). Coupling constants (*J*) are expressed in Hz. LRMS values shown in the synthetic sections were measured by JMS-HX110 spectrometer (JEOL, Tokyo, Japan) and spectroscopic data were recorded as *m*/*z* values.

### 3.2. The Synthetic Procedures for ***5***, ***6***, ***7***, ***8***, ***1-TFA*** and ***2-TFA***

#### 3.2.1. Tert-Butyl (S)-(1-((2-Hydroxyethyl)Amino)-3-Methyl-1-Oxobutan-2-yl)Carbamate (**5**)

To a stirred mixture of *(2S)-2-(tert-butoxycarbonylamino)-3-methyl-butanoic acid* (**3**) (4.000 g, 18.4 mmol), *2-aminoethanol* (**4**) (1.10 mL, 18.4 mmol) and *N,N,N′,N′-tetramethyl-S-(1-oxido-2-pyridyl)thiuronium hexafluorophosphate* (HOTT) (13.663 g, 36.8 mmol) in CH_3_CN (180 mL) was added triethylamine (2.60 mL, 18.4 mmol) at room temperature. The resulting mixture was stirred at the same temperature for 23 h and then concentrated to dryness under vacuo. The crude product was purified by chromatography with ethyl acetate to get the *tert-butyl (S)-(1-((2-hydroxyethyl)amino)-3-methyl-1-oxobutan-2-yl)carbamate* (**5**) (3.930 g, 82%) as a colorless oil.

^1^H NMR (500 MHz, CDCl_3_): 5.54 (br s, 1H), 4.00 (br s, 1H), 3.87 (t, *J* = 7.5 Hz, 1H), 3.63–3.60 (m, 2H), 3.37–3.28 (m, 2H), 1.99–1.97 (m, 1H), 1.36 (s, 9H), 0.90–0.86 (m, 6H); ^13^C NMR (500 MHz, CDCl_3_): 172.9, 156.2, 79.8, 61.4, 60.2, 42.1, 31.0, 28.3, 19.2, 18.1; LRMS [ES]^+^ calculated for C_12_H_24_NaN_2_O_4_: 283.16 [M + Na]^+^; found: 283.25; HRMS [ESI]^+^ calculated for C_12_H_24_NaN_2_O_4_: 283.1634 [M + Na]^+^; found: 283.1640.

#### 3.2.2. Tert-Butyl (S)-(3-Methyl-1-((2-(((4-Nitrophenoxy)Carbonyl)Oxy)Ethyl)Amino)-1-Oxobutan-2-yl)Carbamate (**6**)

To a stirred solution of *tert-butyl (S)-(1-((2-hydroxyethyl)amino)-3-methyl-1-oxobutan-2-yl)carbamate* (**5**) (4.815 g, 18.5 mmol) and *4-nitrophenyl chloroformate* (4.095 g, 20.3 mmol) in CH_2_Cl_2_ (90 mL) was added *N,N-diisopropylethylamine* (DIPEA) (4.756 g, 36.8 mmol) at room temperature. The resulting mixture was stirred at the same temperature for 4 h and then concentrated to dryness under vacuo. The crude residue was purified by chromatography (ethyl acetate/*n*-hexane = 3:2) to provide compound **6** (6.805 g, 87%) as a white solid.

^1^H NMR (500 MHz, CDCl_3_): 8.27 (d, *J* = 9.0 Hz, 2H), 7.40 (d, *J* = 9.0 Hz, 2H), 6.54 (br s, 1H), 5.06 (br s, 1H), 4.35 (t, *J* = 5.5 Hz, 2H), 3.92–3.89 (m, 1H), 3.69–3.62 (m, 2H), 2.14–2.11 (m, 1H), 1.43 (s, 9H), 0.95 (d, *J* = 6.5 Hz, 3H), 0.93 (d, *J* = 6.5 Hz, 3H); ^13^C NMR (500 MHz, CDCl_3_): 172.2, 156.0, 155.4, 152.4, 145.5, 125.3, 121.7, 80.2, 67.8, 60.4, 42.1, 38.2, 30.6, 28.3, 19.2; LRMS [ES]^+^ calculated for C_19_H_27_N_3_NaO_8_: 448.17 [M + Na]^+^; found: 448.49; HRMS [ESI]^+^ calculated for C_19_H_27_N_3_NaO_8_: 448.1696 [M + Na]^+^; found: 448.1686.

#### 3.2.3. Compound **7**

To a mixture of **DMCU** (300 mg, 0.76 mmol) and cesium carbonate (222 mg, 0.68 mmol) in DMF (2 mL) was added *tert-butyl (S)-(3-methyl-1-((2-(((4-nitrophenoxy)carbonyl)oxy)ethyl)amino)-1-oxobutan-2-yl)carbamate* (**6**) (290 mg, 0.68 mmol) in DMF (3 mL). The reaction mixture was stirred at room temperature for 3.5 h and then quenched with quenched with saturated NH_4_Cl_(aq)_. The aqueous layer was separated and extracted with EtOAc (3 × 8 mL). The combined organic extracts were washed with brine, dried over MgSO_4_, filtered and concentrated to give the crude product, which was purified on silica gel with ethyl acetate/*n*-hexane (3:2) to afford **7** (250 mg, 0.37 mmol, 48%) as a yellow solid. ^1^H NMR (DMSO-*d*_6_, 500 MHz)δ 7.52 (d, *J* = 15.6 Hz, 2H), 7.44 (s, 2H), 7.30 (d, *J* = 8.2 Hz, 2H), 7.08–7.04 (m, 4H), 5.16 (br s, 6H), 4.19 (s, 4H), 3.78 (s, 6H), 3.61 (s, 8H), 1.17 (s, 6H); LRMS [ESI]^+^ calculated for C_33_H_40_O_14_: 661.25 [M + H]^+^; found: 661.4.

#### 3.2.4. Compound **8**

To a stirred mixture of **DMCU** (303 mg, 0.76 mmol) and cesium carbonate (493 mg, 1.5 mmol) in DMF (2 mL) was added *tert-butyl (S)-(3-methyl-1-((2-(((4-nitrophenoxy)carbonyl)oxy)ethyl)amino)-1-oxobutan-2-yl)carbamate* (**6**) (888 mg, 2.1 mmol) at room temperature. The resulting mixture was stirred at the same temperature for 6 h and then quenched with saturated NH_4_Cl_(aq)_ (5 mL). The aqueous layer was separated and extracted with EtOAc (3 × 8 mL). The combined organic extracts were washed with brine, dried over MgSO_4_, filtered and concentrated to give the crude product, which was purified on silica gel with EA/*n*-hexane (3:2) to afford **8** (500 mg, 68%) as a yellow solid.

^1^H NMR (400 MHz, CDCl_3_): 7.68 (d, *J* = 15.6 Hz, 2H), 7.13–7.07 (m, 6H), 6.70 (d, *J* = 15.6 Hz, 2H), 6.35 (br s, 1H), 5.02 (br s, 1H), 4.31 (t, *J* = 5.2 Hz, 4H), 3.92–3.90 (m, 2H), 3.87 (s, 6H), 3.69–3.62 (m, 4H), 2.17–2.12 (m, 2H), 1.48 (s, 6H), 1.43 (s, 18H), 0.96 (d, *J* = 7.2 Hz, 6H), 0.91 (d, *J* = 6.4 Hz, 6H); LRMS [ES]^+^ calculated for C_49_H_69_N_4_O_16_: 969.47 [M + H]^+^; found: 970.06.

#### 3.2.5. Compound **1-TFA**

To a stirred solution of **7** (260 mg, 0.38 mmol) in CH_2_Cl_2_ (8 mL) was added trifluoroacetic acid (1.5 mL, 20.0 mmol) at 0 °C. The reaction mixture was warmed to room temperature and stirred for 4 h. The reaction medium was removed under vacuo to give the crude product, which was washed with CH_2_Cl_2_ (2 mL × 3) and concentrated to dryness under vacuo to get the **1-TFA** (264 mg, quantitative yield, 95% purity based on LC analysis) as a yellow solid.

^1^H NMR (400 MHz, CD_3_OD): 7.70–7.64 (m, 2H), 7.33 (d, *J* = 1.6 Hz, 1H), 7.24–7.10 (m, 4H), 6.96 (d, *J* = 15.6 Hz, 1H), 6.84–6.79 (m, 2H), 4.38–4.26 (m, 2H), 3.87 (s, 3H), 3.86 (s, 3H), 3.74–3.67 (m, 1H), 3. 63 (d, *J* = 5.6 Hz, 1H), 3.53–3.47 (m, 1H), 2.22–2.13 (m, 1H), 1.48 (s, 6H), 1.08–1.04 (m, 6 H). ^13^C NMR (125 MHz, *d*-DMSO): 199.1, 198.8, 168.6, 158.8 (q, *J* = 40.0 Hz), 152.7, 151.5, 150.1, 148.3, 144.4, 142.4, 141.4, 134.0, 126.1, 124.1, 123.2, 122.1, 119.0, 116.1, 113.1, 112.2, 67.5, 59.9, 58.1, 56.6., 56.2, 38.1, 31.3, 30.1, 22.4, 21.3, 18.5, 18.1, 14.3; LRMS [ES]^+^ calculated for C_31_H_39_N_2_O_9_: 583.26 [M + H]^+^; found: 583.72; HRMS [ESI]^+^ calculated for C_31_H_39_N_2_O_9_: 583.2650 [M + H]^+^; found: 583.2651.

#### 3.2.6. Compound **2-TFA**

To a solution of **8** (316 mg, 0.33 mmol) in CH_2_Cl_2_ (10 mL) was added trifluoroacetic acid (3 mL, 39.2 mmol) at 0 °C. The reaction mixture was warmed to room temperature and stirred for 4 h. The reaction medium was removed under vacuo to give the crude product, which was washed with CH_2_Cl_2_ (2 mL × 3) and concentrated to dryness under vacuo to get the **2-TFA** (323 mg, quantitative yield, 95.8% purity based on LC analysis) as a yellow solid.

^1^H NMR (400 MHz, CD_3_OD): 7.69 (d, *J* = 15.6 Hz, 2H), 7.35 (d, *J* = 2.0 Hz, 2H), 7.26–7.23 (m, 2H), 7.14 (d, *J* = 8.0 Hz, 2H), 7.01 (d, *J* = 15.6 Hz, 2H), 4.38–4.26 (m, 4H), 3.87 (s, 6H), 3.74–3.68 (m, 2H), 3.64 (d, *J* = 5.2 Hz, 2H), 3.53–3.47 (m, 2H), 2.22–2.14 (m, 2H), 1.50 (s, 6H), 1.08–1.05 (m, 12H); ^13^C NMR (125 MHz, *d*-DMSO): 199.2, 168.6, 158.8 (q, *J* = 41.2 Hz), 152.7, 151.5, 142.6, 141.4, 134.0, 123.2, 123.0, 122.6, 113.1, 67.5, 60.0, 58.1, 56.5, 38.1, 30.1, 21.1, 18.5, 18.1; LRMS [ES]^+^ calculated for C_39_H_54_N_4_O_12_: 385.18 [M + 2H]^2+^; found: 385.94; HRMS [ESI]^+^ calculated for C_39_H_54_N_4_O_12_: 385.1863 [M + 2H]^2+^; found: 385.1864

### 3.3. Biological Assays

#### 3.3.1. Cell Culture

Human NSCLC cell lines (NCI-H460, NCI-H358 and A549) were obtained from ATCC. All cell lines were grown in RPMI medium supplemented with 10% fetal bovine serum (FBS) and antibiotics and incubated at 37 °C in a humidified atmosphere with 5% CO_2_.

#### 3.3.2. In Vitro Antiproliferative Assay

Antiproliferative activities to various compounds was assessed using MTT assay [31]. In brief, 2000–5000 cells were seeded in each well of 96-well plates and incubated at 37 °C overnight before drug treatment. After a three-day drug treatment, MTT solution was added to each well and the plates were further incubated at 37 °C for 6 h. Measurement of cell viability was assessed by absorbance at 570 nm. The IC_50_ values for each drug were determined using CompuSyn software based on the median-effect principle and plot.

### 3.4. Preparation of Standard Solution of ***DMCU***, ***1-TFA*** and ***2-TFA***

The stock solutions of **1-TFA** and **2-TFA** were prepared by dissolving 20 mg of **1-TFA** and **2-TFA**, respectively, in 1 mL of 1% formic acid_(aq)_. The standard solution of **1-TFA** or **2-TFA** of 0.5, 1.25, 2.5, 5 and 12.5 μg/mL was prepared by diluting the aforementioned stock solution with an adequate amount of 40% methanol in ultrapure water. The stock solution of **DMCU** was prepared by dissolving 10 mg of **DMCU** in 1 mL of methanol. The **DMCU** stock solution was diluted with 40% methanol in ultrapure water to afford three standard solutions with concentrations of 0.08, 0.4, and 2 μg/mL.

### 3.5. Preparation of Aqueous Solution of ***1-TFA*** and ***2-TFA*** for Solubility Analysis

The saturated aqueous solutions of compound **1-TFA** and **2-TFA** were prepared by a three-steps sequence including supersaturated solution preparation (addition of 20 mg compounds into 50 μL ultrapure water), centrifugation (10 min at 14,000× *g*), and collection of the resulting supernatants to afford the desired solutions. The saturated aqueous solutions were then diluted by ultrapure water as the ratio of 1 to 100,000 for LC-MS analysis.

### 3.6. Analysis Method of LC-MS

The LC-MS system consisted of an ultraperformance liquid chromatography (UPLC) system (ACQUITY UPLC I-Class, Waters, Milford, MA, USA and an ESI/APCI source of 4 kDa quadrupole time-of-flight mass spectrometer (Waters VION, Waters, Milford, MA, USA). A BEH C18 column (2.1 × 100 mm, Walters, Milford, MA, USA) was equipped to perform chromatographic separation. The flow rate of 0.2 mL/min with a column temperature of 35 °C was set for each sample injection. The elution started with 60% mobile phase A (ultrapure water + 0.1% formic acid) and 40% mobile phase B (100% methanol + 0.1% formic acid), then held at 40% B for 0.5 min, raised to 95% B in 5.5 min, held at 95% B for 1 min, and then lowered to 40% B in 1 min. The column was equilibrated by pumping 40% B for 4 min. The LC-MS data were acquired using ESI+ mode under the following conditions: capillary voltage of 2.5 kV, source temperature of 100 °C, desolation temperature of 250 °C, cone gas maintained at 10 L/h, desolation gas maintained at 600 L/h, and acquisition by MS^E^ mode with a range of 100–1000 *m*/*z* and a 0.5-s scan time. The data were acquired and processed using UNIFI software (Waters, Milford, MA, USA).

### 3.7. Cell Extracts for LC-MS Analysis

NCI-H460 cells, inoculated in the 6-well plates, were maintained at 37 °C for 24 h and treated with 10 μM of **DMCU**, **1-TFA** or **2-TFA**. The cells were cultured at 37 °C for 0.5, 1, 2 and 4 h, respectively. For each well, the used medium was removed and the residual cells were washed with 1 mL of fresh PBS three times. After completely removing the residual solution, 100 μL of ultrapure water was added and vigorous pipetting was performed to disrupt the cells. After centrifuging at 14,000× *g* for 10 min, 40 all of supernatant from cell homogenate was collected and then mixed with 160 μL of methanol. The resulting mixture was stored at −80 °C and used as an aliquot of cell extract. For LC-MS analysis, the frozen cell extract was warmed to room temperature and centrifuged at 14,000× *g* for 10 min to provide the supernatant, which was used directly for sample injection.

## 4. Conclusions

Here, we have developed two **DMCU** derivatives, **1-TFA** and **2-TFA**, as effective anticancer agents against NSCLCs with LAT-1 expression. The present study provides a direction for future structural modification of curcuminoid-like phenolic compounds. Additional works on the changes of L-valine into other BCAAs have been scheduled for enhancement of cellular uptake in lung cancer cells. For the evaluation of drug uptake, an alternative estimation method based on the LC-MS was established and revealed that modification of **DMCU** into **2**-**TFA** facilitated the cell uptake. Such outcomes are in line with the superior anticancer activity of **2**-**TFA**. However, the factors that caused the disparities between the antiproliferative effects and cellular uptake abilities in the **DMCU** and **1-TFA** treated groups are still unclear. Further evaluations and mechanism studies will be performed in the future.

## Figures and Tables

**Table 1:** The anti-proliferative effects of DMCU, 1-TFA, and 2-TFA against NCI-H460, NCI-H358, and A549 lung cancer cell lines.

Compound	IC_50_ ^a,b^ (μM)		
	NCI-H460	NCI-H358	A549
DMCU	2.13 ± 0.16	1.62 ± 0.12	2.50 ± 0.03
1-TFA	1.95 ± 0.16	1.92 ± 0.13	3.17 ± 0.14
2-TFA	1.69 ± 0.04	1.26 ± 0.06	1.99 ± 0.24

^a^. NCI-H460, NCI-H358 and A549 cancer cells were treated with different concentration of compounds for 72 h. ^b^. Data are presented as mean ± SD.

## Data Availability

The data present in this study are available on the request from the corresponding authors.

## Supplementary Materials

The following are available online. Copies of 1H, 13C NMR and mass spectra of the synthetic products (**5**, **6**, **7**, **8**, **1-TFA** and **2-TFA**).
