# Supplementary material for: Synthesis and Characterization of the Ethylene-Carbonate-Linked L-Valine Derivatives of 4,4-Dimethylcurcumin with Potential Anticancer Activities
Source: Molecules. 2021 Nov 22;26(22):7050. doi: 10.3390/molecules26227050 (PMC8624457; doi:10.3390/molecules26227050)
Supplement: Supplementary file 1 [file molecules-26-07050-s001.zip › molecules-1474745-supplementary.pdf]

Supporting Information

Synthesis and characterization of the ethylene-carbonate-linked L-valine derivatives of 4,4-dimethylcurcumin with potential anticancer activities

Der-Yen Lee, Hui-Yi Lin, Manickavasakam Ramasamy, Sheng-Chu Kuo, Pei-Chih Lee and Min-Tsang Hsieh

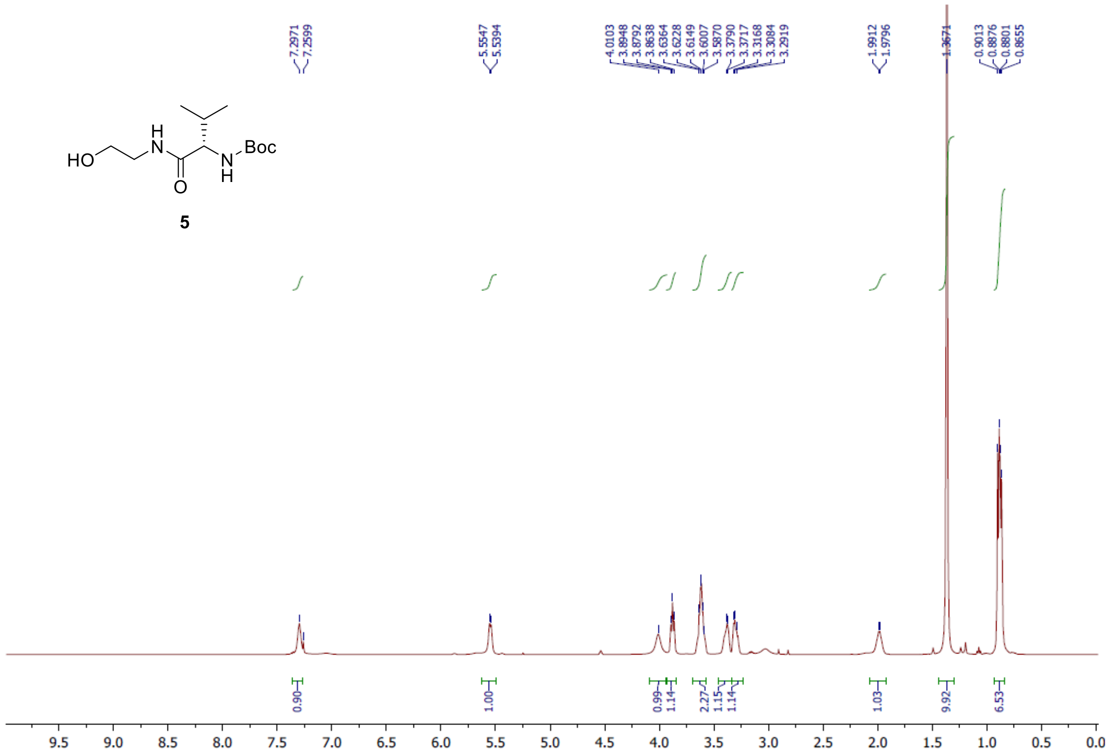

^{1}H spectrum of **5**

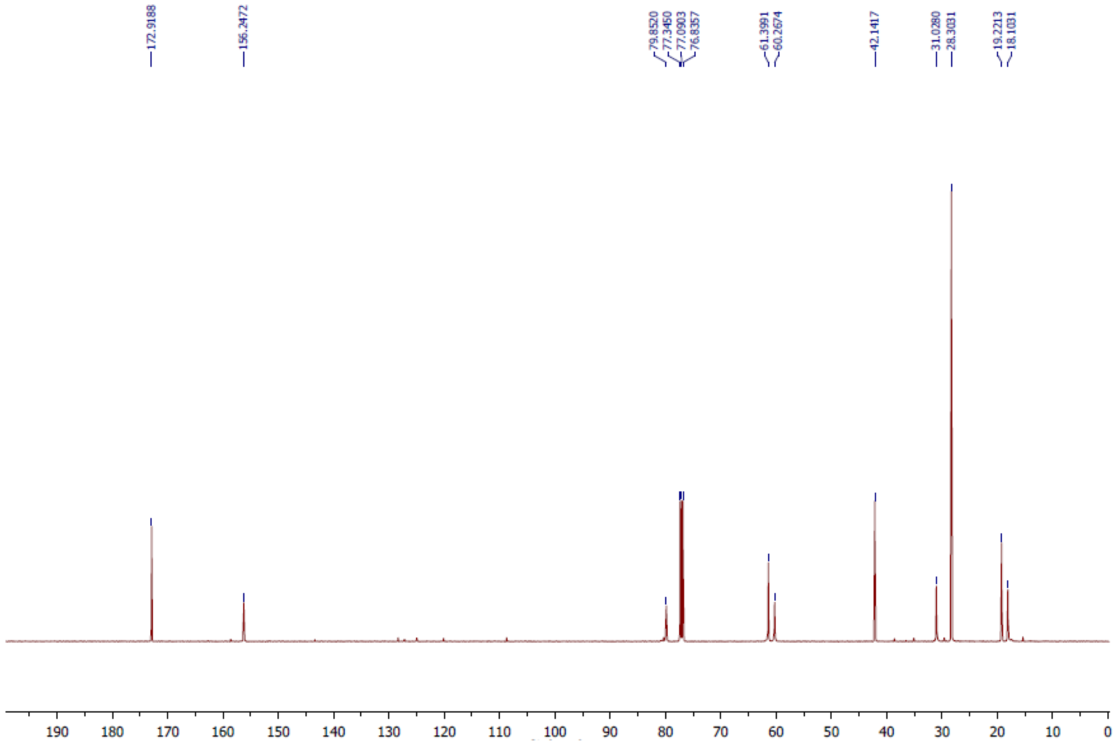

^{13}C spectrum of **5**

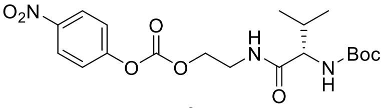

6

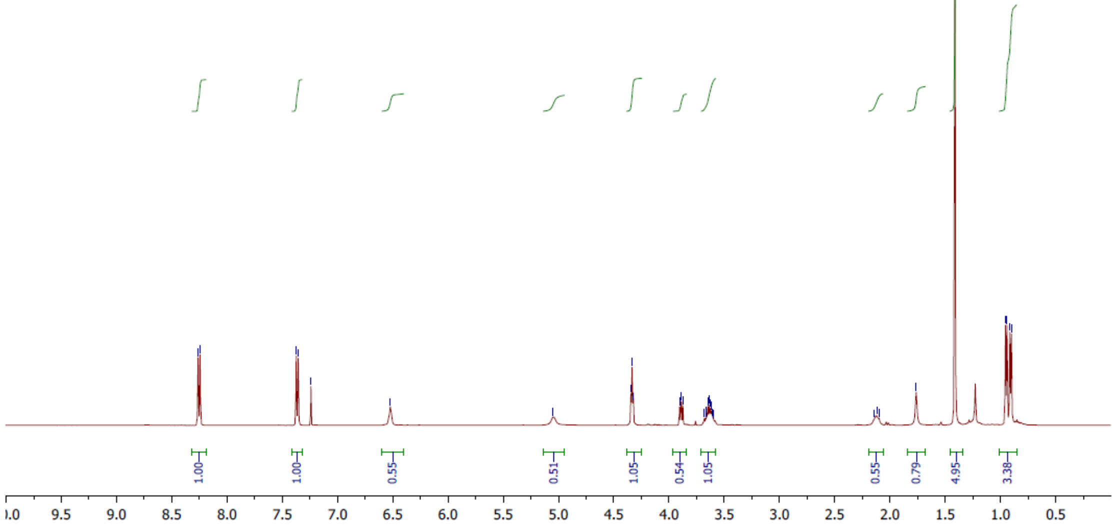

¹H spectrum of **6**

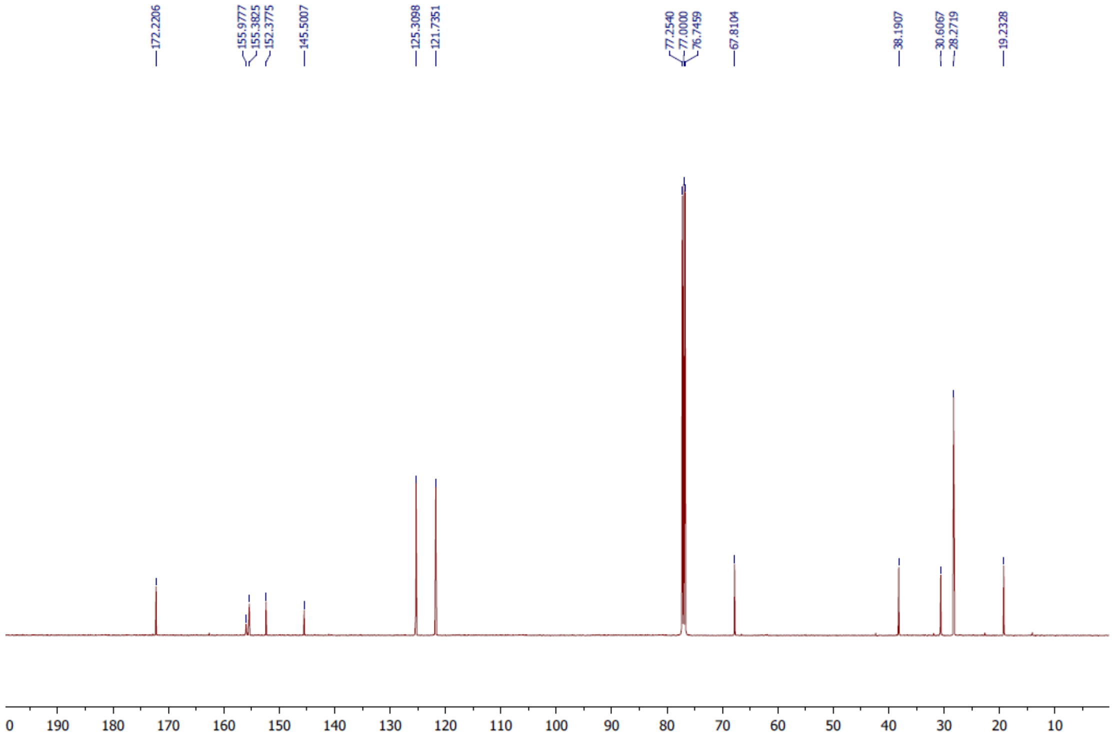

¹³C spectrum of **6**

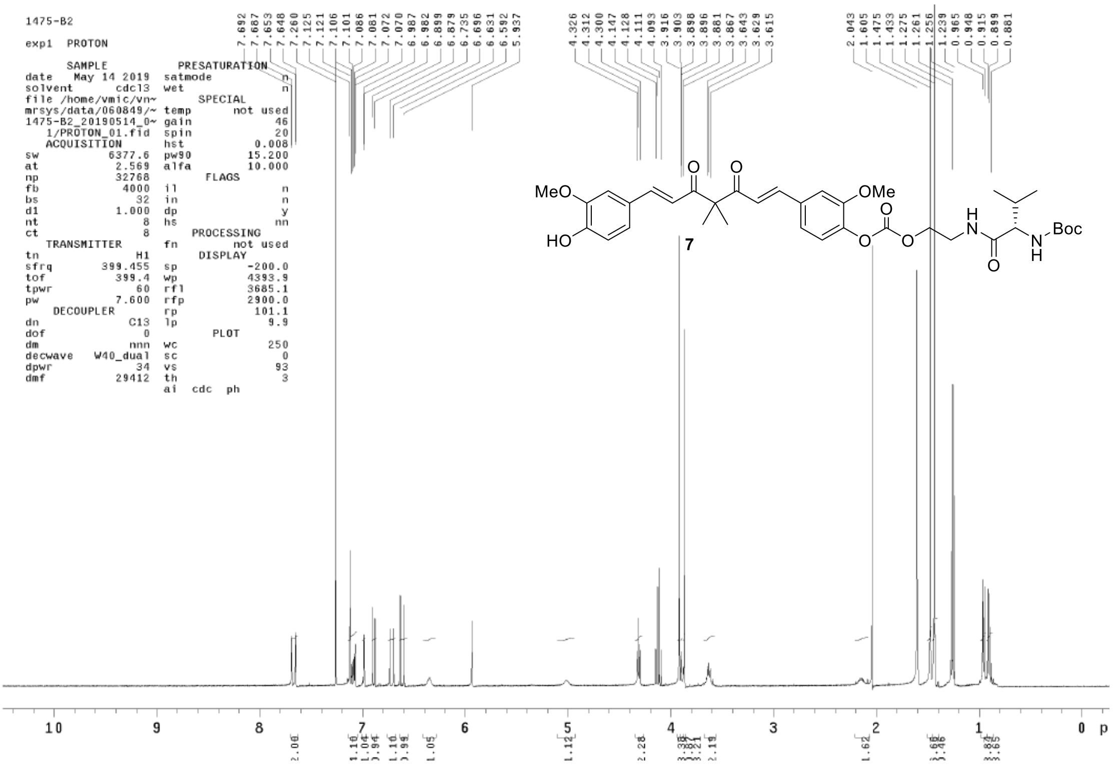

^{1}H spectrum of **7**

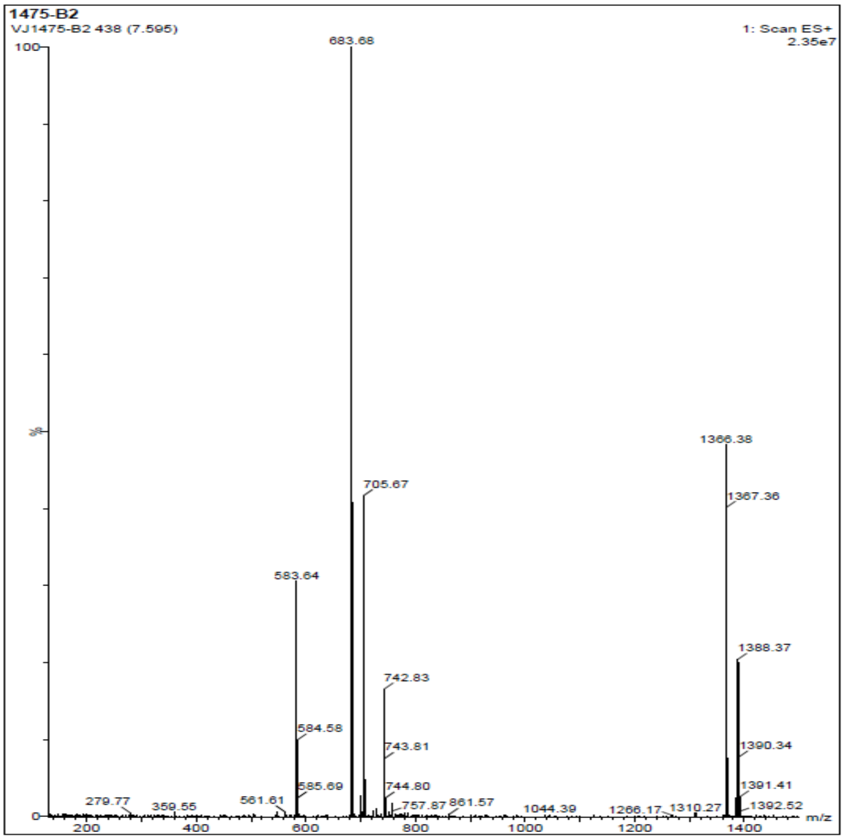

Mass spectrum of **7**

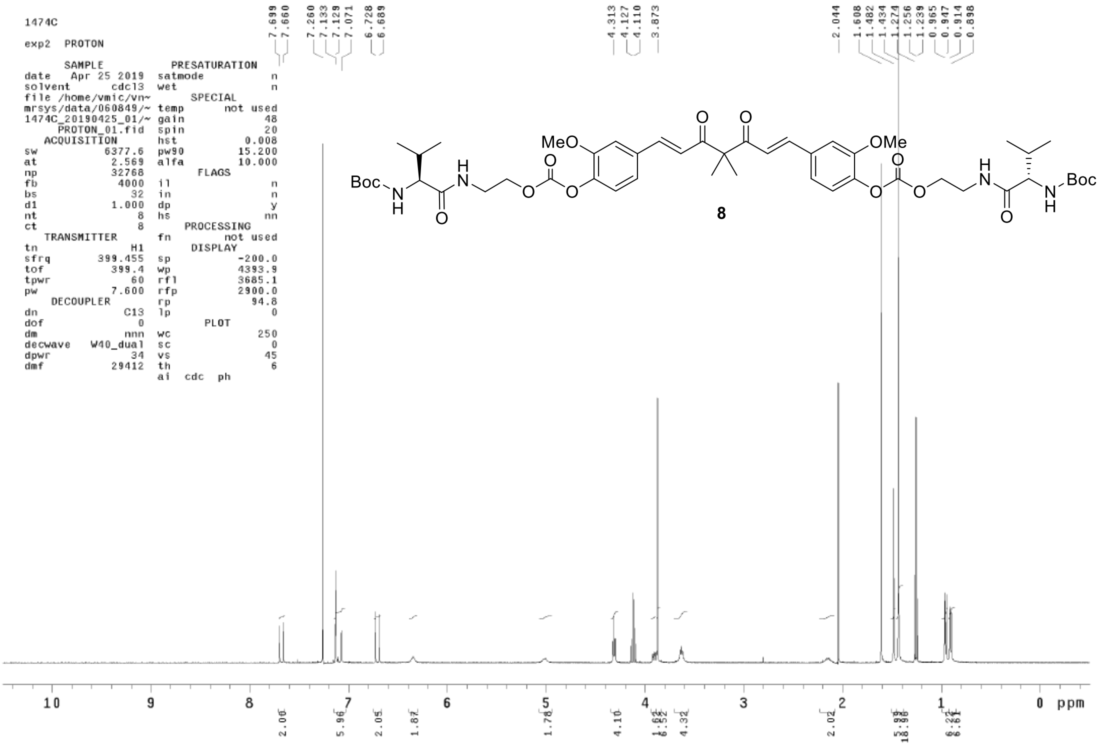

^{1}H spectrum of **8**

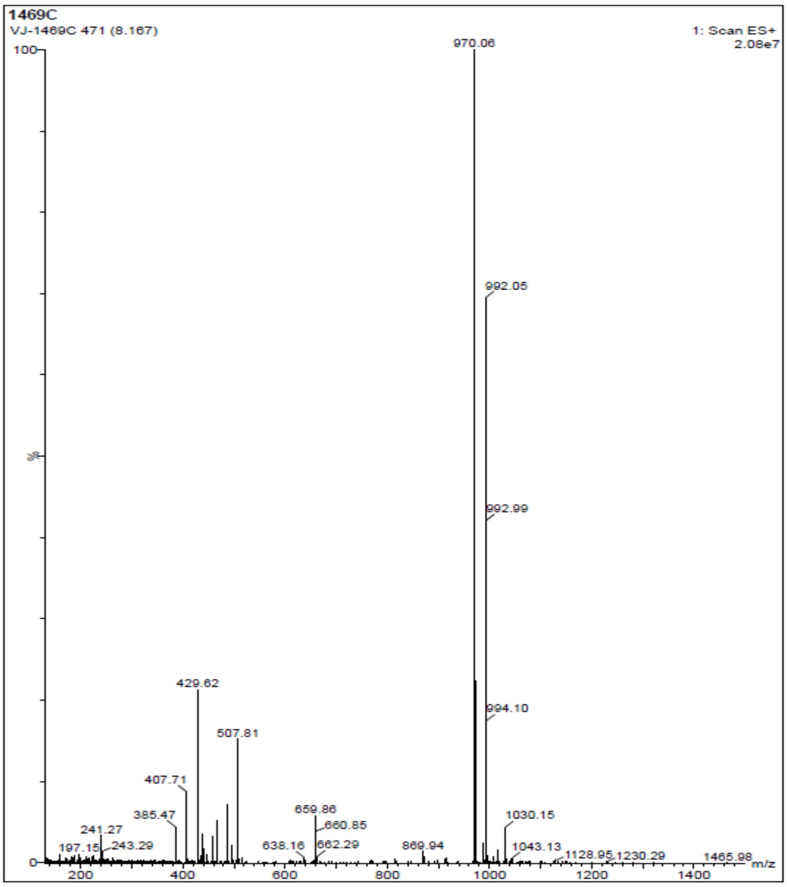

Mass spectrum of **8**

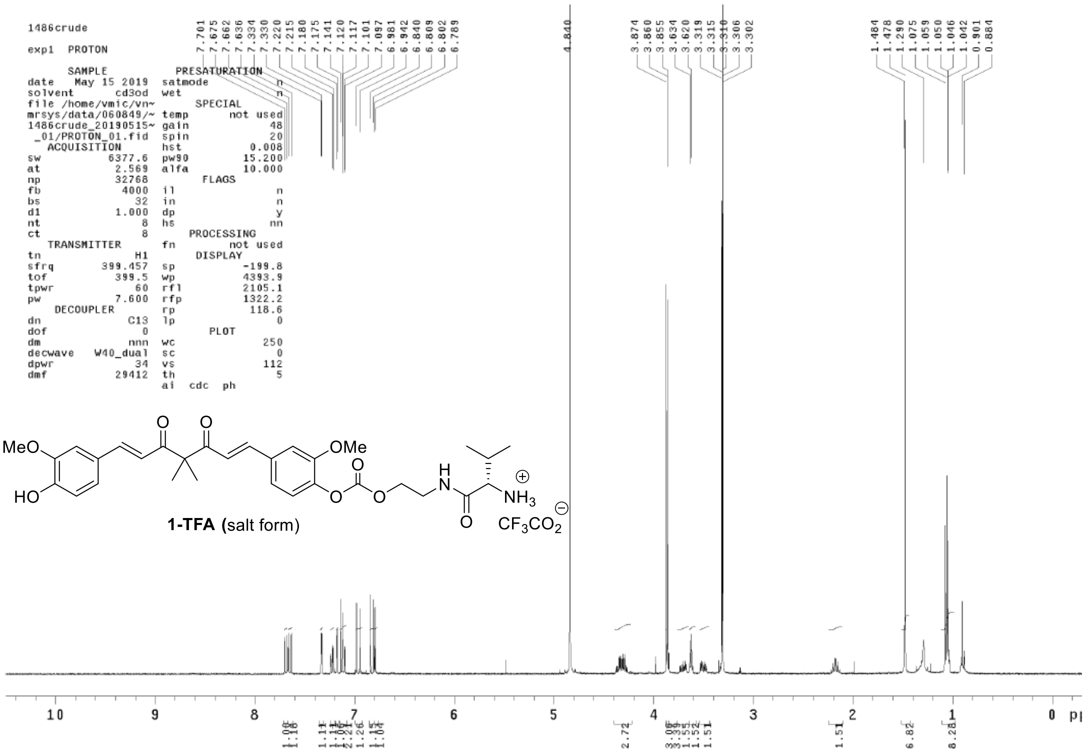

^{1}H spectrum of **1-TFA**

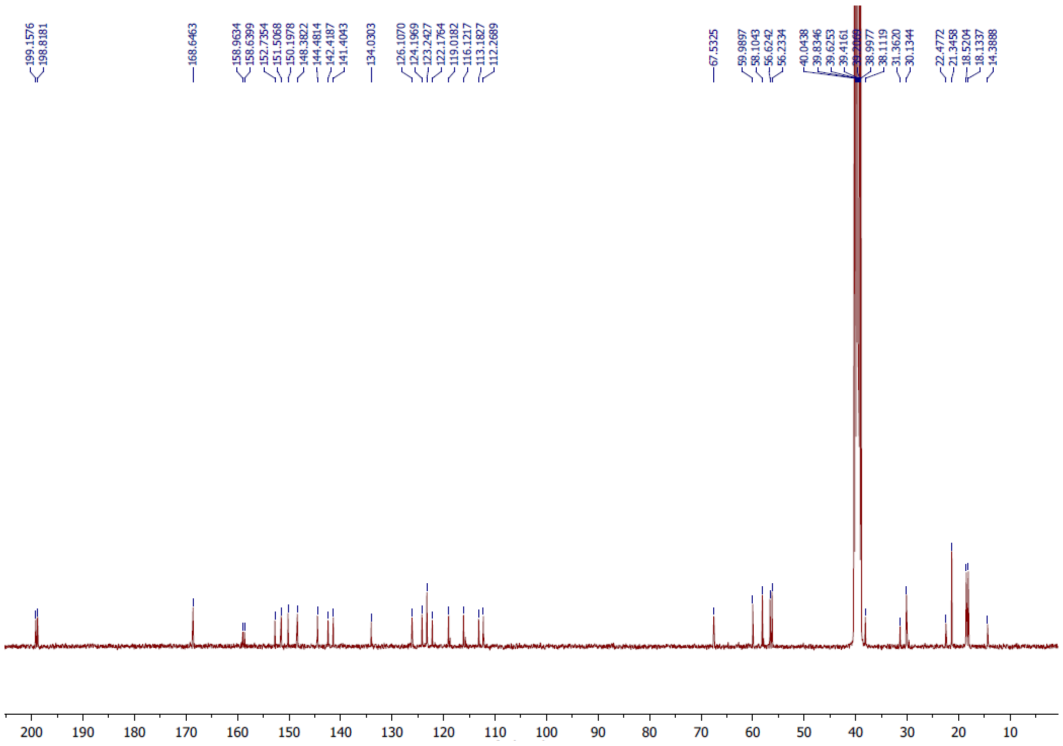

^{13}C spectrum of **1-TFA**

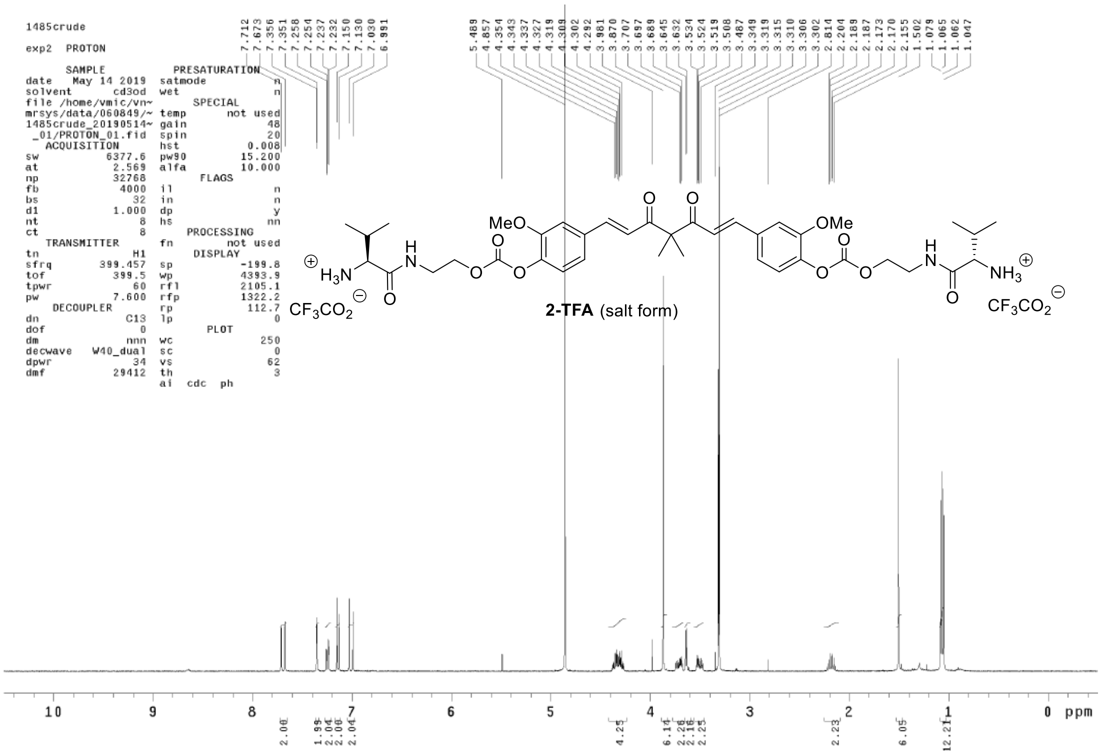

^{1}H spectrum of **2TFA**

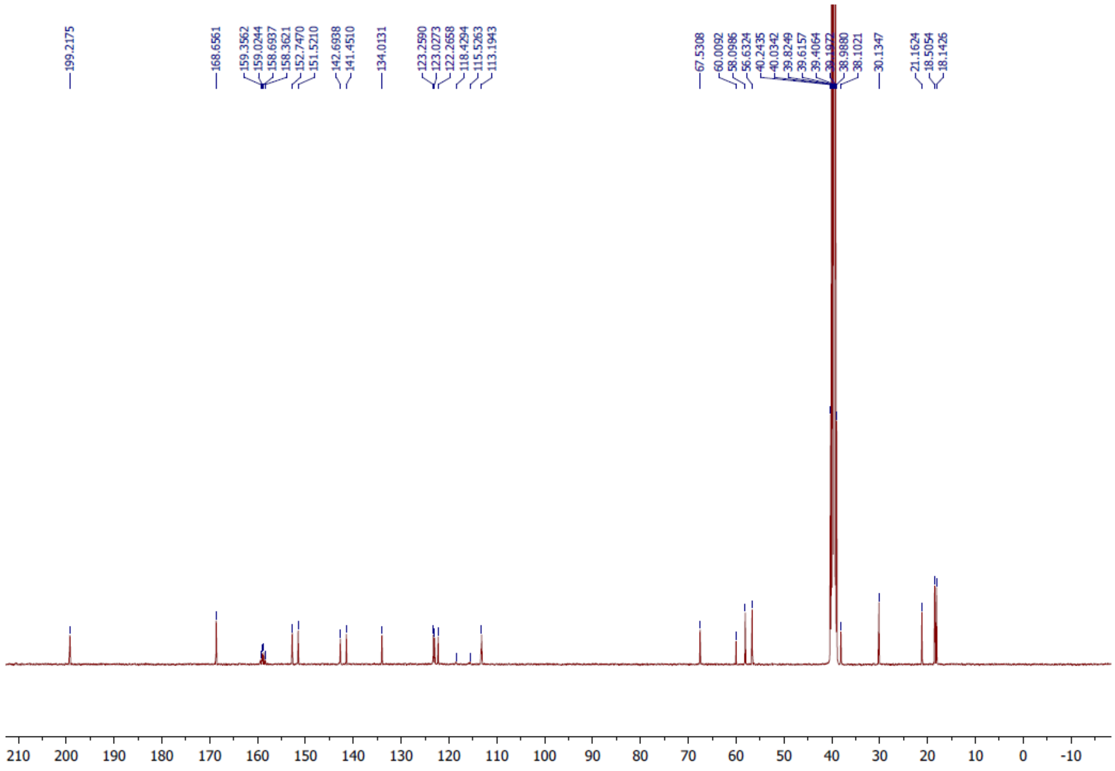

^{13}C spectrum of **2TFA**
